# Transmission of *Leishmania infantum* in the Canine Leishmaniasis Focus of Mont-Rolland, Senegal: Ecological, Parasitological and Molecular Evidence for a Possible Role of *Sergentomyia* Sand Flies

**DOI:** 10.1371/journal.pntd.0004940

**Published:** 2016-11-02

**Authors:** Massila Wagué Senghor, Abdoul Aziz Niang, Jérome Depaquit, Hubert Ferté, Malick Ndao Faye, Eric Elguero, Oumar Gaye, Bulent Alten, Utku Perktas, Cécile Cassan, Babacar Faye, Anne-Laure Bañuls

**Affiliations:** 1 Laboratory of Terrestrial Invertebratede Zoology, IFAN (Institut Fondamental d'Afrique Noire), Université Cheikh Anta Diop, Dakar, Senegal; 2 MIVEGEC (UMR IRD 224-CNRS 5290- Université Montpellier), Montpellier, France; 3 Laboratory of Parasitology Mycology, Faculty of Medicine, Pharmacy and Odonto-Stomatology, University Cheikh Anta Diop, Dakar, Senegal; 4 Université de Reims Champagne-Ardenne, ANSES, EA4688 - USC «transmission vectorielle et épidémiosurveillance de maladies parasitaires (VECPAR)», SFR Cap Santé, Faculté de Pharmacie, Reims, France; 5 Department of Biology (Zoology Section) Faculty of Science, Hacettepe University, Beytepe - Ankara, Turkey; National Institutes of Health, UNITED STATES

## Abstract

*Leishmania* (*L*.) *infantum* is the causative agent in an endemic focus of canine leishmaniasis in the Mont-Rolland district (Thiès, Senegal). In this area, the transmission cycle is well established and more than 30% of dogs and 20% of humans are seropositive for *L*. *infantum*. However, the sand fly species involved in *L*. *infantum* transmission cycle are still unknown. Between 2007 and 2010, 3654 sand flies were collected from different environments (indoor, peridomestic, farming and sylvatic areas) to identify the main *L*. *infantum* vector(s). Nine sand fly species were identified. The *Phlebotomus* genus (n = 54 specimens; *Phlebotomus* (*Ph*) *duboscqi* and *Phlebotomus* (*Ph*). *rodhaini*) was markedly under-represented in comparison to the *Sergentomyia* genus (n = 3600 specimens; *Sergentomyia* (*Se*) *adleri*, *Se*. *clydei*, *Se*. *antennata*, *Se*. *buxtoni*, *Se*. *dubia*, *Se*. *schwetzi* and *Se*. *magna*). *Se*. *dubia* and *Se*. *schwetzi* were the dominant species indoor and in peridomestic environments, near humans and dogs. Blood-meal analysis indicated their anthropophilic behavior. Some *Se*. *schwetzi* specimens fed also on dogs. The dissection of females in the field allowed isolating *L*. *infantum* from sand flies of the *Sergentomyia* genus (0.4% of *Se*. *dubia* and 0.79% of *Se*. *schwetzi* females). It is worth noting that one *Se*. *dubia* female not engorged and not gravid revealed highly motile metacyclic of *L*. *infantum* in the anterior part of the midgut. PCR-based diagnosis and sequencing targeting *Leishmania* kinetoplast DNA (kDNA) highlighted a high rate of *L*. *infantum*-positive females (5.38% of *Se*. *dubia*, 4.19% of *Se*. *schwetzi* and 3.64% of *Se*. *magna*). More than 2% of these positive females were unfed, suggesting the parasite survival after blood-meal digestion or egg laying. *L*. *infantum* prevalence in *Se*. *schwetzi* was associated with its seroprevalence in dogs and humans and *L*. *infantum* prevalence in *Se*. *dubia* was associated with its seroprevalence in humans. These evidences altogether strongly suggest that species of the *Sergentomyia* genus are probably the vectors of canine leishmaniasis in the Mont-Rolland area and challenge one more time the dogma that in the Old World, leishmaniasis is exclusively transmitted by species of the *Phlebotomus genus*.

## Author Summary

Leishmaniases, neglected tropical vector-borne diseases, remain today a problem of public health. Classically, the sand flies involved in *Leishmania* transmission belong either to the *Phlebotomus* genus (Old World) or to the *Lutzomyia* genus (New World). In the rural community of Mont-Rolland (Senegal, West Africa), *Leishmania infantum* is the causative agent in an endemic focus of canine leishmaniasis. Recent surveys revealed more than 30% of dogs and 20% of humans with a positive serological test for *Leishmania* in this community. However, the sand fly species involved in *L*. *infantum* transmission were still unknown. Between 2007 and 2010, we carried out a study in this community to identify the sand fly species responsible for *L*. *infantum* transmission. We collected nine species belonging mainly to *Sergentomyia* genus and in low proportion to *Phlebotomus* genus. The abundance around dogs and humans, the detection of live and mature parasites in anterior midgut, the high rate of *L*. *infantum*-positive females using molecular analyses and the identification of dog and human blood in the fed females incriminates *Se*. *schwetzi* and *Se*. *dubia* as possible vectors of *L*. *infantum*. This hypothesis is strongly supported by statistical analyses performed to compare the prevalence of infected sand flies with the seroprevalence data in humans and dogs.

## Introduction

Leishmaniases are vector-borne diseases with complex ecology and epidemiology. In humans, they are caused by more than 20 species of *Leishmania* parasites that live in a wide range of ecosystems and may have different clinical manifestations (mainly cutaneous, mucocutaneous or visceral) [1]. It is classically acknowledged that the sand flies involved in *Leishmania* transmission belong to the genus *Phlebotomus* in the Old World and to the genus *Lutzomyia* (sensu Young & Duncan) in the New World [2, 3].

In the rural community of Mont-Rolland (Senegal, West Africa), *L*. *infantum* is the causative agent in an endemic focus of canine leishmaniasis described since 1970 [4, 5]. More recent studies have clearly shown that *L*. *infantum* circulation is well established in this focus and more than 30% of dogs and 20% of humans have a positive serologic test result [6]. However, to our knowledge, only one human clinical case has been recorded: one child with several cutaneous lesions [6]. This *L*. *infantum* canine leishmaniasis focus is particularly interesting because of its unusual location (i.e., outside the Mediterranean basin, Central and Southwest Asia, China, Middle East, Central and South America etc.) [7, 8] and because of the absence of the usual vectors (*Phlebotomus* sand flies) for this parasite.

Therefore, the main objectives of this study were to identify the vector(s) of *L*. *infantum* and to describe the transmission cycle in Mont-Rolland. Sand flies were caught in various environments (indoor, peridomestic, farming and sylvatic areas) to determine their degree of endophily and exophily. The presence of *Leishmania* promastigotes was investigated using a classical parasitological method (dissection under the microscope and culture) and/or by PCR detection of *Leishmania* kinetoplast DNA (kDNA) and sequencing. The physiological status of females (engorged, gravid or unfed) and the blood-meal origin in blood-fed females were also determined. Finally, the association of PCR-positive specimens with the rate of infected dogs and seropositive humans was investigated.

## Material and Methods

### Studied area

**Fig 1:**
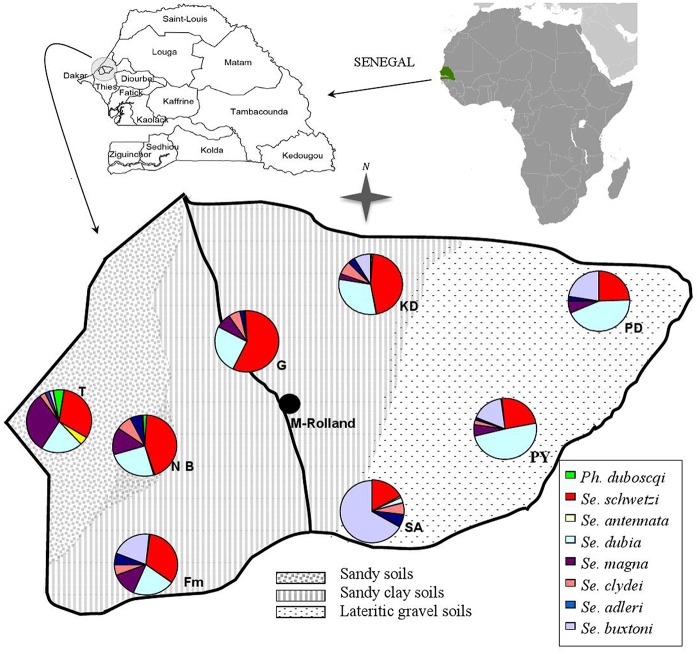
Map describing the Mont-Rolland community and the distribution of sand fly species in the different villages. Map of the **Mont-Rolland** community situated in the Thiès region, Senegal. Identification of the sylvatic area (SA) and the seven villages selected for sample collection: Fouloum (Fm), Guidieur (G), Khaye Diagal (KaD), Ndiaye Bopp (NB), Pallo Youga (PY), Pallo Dial (PD) and Thiaye (T). The soil characteristics and the distribution of the different species of phlebotomine sand flies in each village and in the sylvatic area are indicated. As *Ph*. *rodhaini* was represented only by eight specimens in three different villages (two in Thiaye, three in Fouloum, two in Khaye Diagal and one in Ndiaye Bopp), they could not be shown on the map.

The rural community of Mont-Rolland (population: 18,000 inhabitants) is located about 15 km north of Thiès city (Western Senegal), at latitudes 14°55’–14°56’N and longitudes 16°50’–16°55’W (Fig 1). The climate is tropical, typical of the Soudan-Sahel region. The rainy season lasts generally from July to October. The annual rainfall in this area is between 500 and 650 mm with an average annual temperature of 26.7°C. The lowest temperatures are recorded during the dry season, with a minimum of 24.4°C, and the highest ones during the rainy season, with a maximum of 29.2°C. Hygrometry presents seasonal and daily variations. The maximum is about 90% relative humidity (RH) during the second half of the night in the rainy season and the minimum is about 25% RH during the day at the end of the dry season [9].

### Sand fly collection and species identification

Sand flies were collected during seven days in April and then in June or July (before the rainy season), each year, from 2007 to 2010. Two types of interception and attraction methods (CDC miniature light trap (John W. Hock Co. FL, U.S.A.), sticky paper) and pyrethroid spraying were used according to the procedures described in Niang et al. [10]. and Abonnenc [11] Sticky traps and CDC light traps were set before sunset and retrieved the following day, early in the morning. Indoor spraying with pyrethroid insecticides was carried out between 7 and 10 am. Collections were carried out in seven villages (Fouloum, Guidieur, Khaye Diagal, Ndiaye Bopp, Pallo Youga, Pallo Dial, and Thiaye) of the Mont-Rolland community (Fig 1), where previous studies reported the presence of a large number of sick dogs [6] and various sand fly species [12]. In each village, sand flies were caught in various environments with different levels of anthropization (indoor, peridomestic, farming and sylvatic areas), according to the following plan:

#### Indoor

Three CDC light traps per village were placed in bedrooms or storerooms.

Indoor spraying was performed in three houses per village.

#### Peridomestic environment

Three CDC light traps per village were placed in henhouses, inside and outside animal shelters and dog kennels.

Ten sticky papers were placed at the entrance of holes and other openings around dwellings.

#### Farming areas (more than 1km from houses)

Three CDC light traps were placed near burrows and/or under trees.

Ten sticky papers were placed in agricultural plantations, rodent burrows and in termite mounds, cracks in the soil, tree trunks.

#### Isolated environment (sylvatic areas)

Three CDC light traps were placed near burrows and/or under trees.

Ten sticky papers were placed in rodent burrows, in termite mounds, cracks in the soil, tree trunks.

Collected sand flies were sorted in the field by environment and by trap for determining the distribution of species and of parasite-positive specimens, their resting behavior, endophily and exophily.

A fast species-identification method was used in this study. The head and the last few abdominal segments were removed from the rest of the body and they were mounted between slide and cover slip after clearing in boiling Marc-André solution. Specimens were then identified morphologically at species level using a photonic force microscope with magnifications ranging from x100 to x400. For identification, the structures of the genitalia and pharyngeal and cibarial armatures were taken into account according to the keys published in Abonnenc, [11] and Niang *et al*. [10].

After species identification, females were processed according to the capture mode and the study objectives (see below), while males were all kept in tubes containing 70% alcohol.

### Detection of Leishmania promastigotes

In 2008 and 2009, female sand flies caught by indoor spraying were dissected in a drop of sterile saline (0.9%) and examined microscopically for the presence of *Leishmania* promastigotes in the digestive tract. Females collected with CDC light traps were kept alive and immobilized with cigarette smoke before dissection. They were then transferred to a sterilized microscope slide in a drop of sterile saline solution (0.9%). The head and genitalia were used for species identification, as detailed above, while the gut was carefully dissected for promastigote detection by microscopic examination. When flagellates were observed in the gut, the cover glass was gently removed and more physiologic solution added. The liquid containing the digestive tract was then aspirated and inoculated in a culture tube containing Novy-MacNeal-Nicolle (NNN) medium with 0.75 ml of penicillin diluted in physiological serum (final concentration: 100,000 UI/ml). Tubes were placed at 26°C and monitored under a microscope after three days and then twice a week for four to six weeks. In positive cultures, the *Leishmania* species was identified by using a nested PCR-based method [13] followed by sequencing. As isolation of promastigotes from female sand flies caught on sticky paper is difficult because they are generally dead for too long (i.e. several hours) and that we could not keep them refrigerated until dissection, these specimens were stored in 70% ethanol and were used for species identification and kDNA detection.

### DNA extraction and Leishmania kDNA detection by PCR

After species identification by microscopic analysis, whole DNA (including kDNA) was extracted from the rest of the body using the Qiagen DNeasy Blood & Tissue Kit. To validate the microscopic identification, *Leishmania* kDNA was amplified by nested PCR, according to Noyes *et al*. [13], to detect the presence of parasites and to differentiate the main Old World *Leishmania* species, particularly *L*. *major*, *L*. *tropica*, *L*. *infantum* and *L*. *tarentolae*. The amplification products were analyzed on 1.6% agarose gels. We used the following reference strains for species identification: *L*. *infantum*, MHOM/MA/67/ITMAP263; *L*. *major*, MHOM/IL/80/Friedlin or MHOM/SU/73/5ASKH; *L*. *tropica*, MHOM/SU/74/K27; *L*. *tarentolae*, RTAR/SN/67/G10. The amplicons obtained from the positive cultures (see above) were then sent to Eurofins MWG Operon for purification and sequencing to confirm the species.

### Blood-meal analysis of engorged females

Blood-fed females collected with sticky paper, light traps and indoor spraying were stored individually in Eppendorf tubes containing 70% ethanol. Females were identified and DNA extraction was carried out as detailed above.

To identify the blood-meal source, first the presence of human DNA was investigated by PCR amplification of the human-specific AluYb8 repeat, according to the procedure described by Deininger and Batzer [14]. In the negative samples, the presence of the mammalian prepronociceptin (*PNOC*) gene was then assessed as described by Haouas *et al*. [15]. Amplicons were then sequenced by Eurofins Genomics. Sequences were identified using BLAST (Basic Local Alignment Search Tool, National Center for Biotechnology Information).

### Statistical analysis

All data collected in this study and the results of the serological studies performed in dogs and humans by Faye *et al*. [9, 16] were used for statistical analyses. The association between each species and the environment was studied by logistic regression, where the response was the proportion of sand flies of the given species among captured sand flies. Global significance was assessed by likelihood ratio tests, and partial Wald tests were used to test the nullity of each estimated parameter. Post-hoc analysis was performed using single-step adjustment of *P*-values. We also measured the relationship between the sand fly infection rate and environment on one hand and *Leishmania infantum* seroprevalence in dogs and humans on the other hand. All computations were carried out with the R software (R-core team 2015) [17] and specifically the *multcomp* package [18].

## Results

### Sand fly species identification and abundance

A total of 3654 sand fly specimens (1070 males and 2584 females) was captured. Microscopic identification showed that sand flies belonging to the *Phlebotomus* genus (54 specimens caught) were much less abundant than those belonging to the *Sergentomyia* genus (3600 specimens caught). Nine species were identified. Two belonged to the *Phlebotomus* genus (*Ph*. *duboscqi* Neuveu-Lemaire, 1906 and *Ph*. *Rodhaini* Parrot, 1930) and the other seven to the *Sergentomyia* genus (*Se*. *schwetzi* Adler, Theodor et Parrot 1929, *Se*. *dubia* Parrot, Mornet et Cadenat, 1945, *Se*. *buxtoni* Theodor, 1933, *Se*. *magna* Sinton, 1932, *Se*. *clydei* Sinton, 1928, *Se*. *adleri Theodor*, 1933 and *Se*. *antennata* Newstead, 1912) (Table 1). Their distribution in the seven villages of the rural community of Mont-Rolland is shown in Fig 1.

**Table 1:** Number of individuals per phlebotomine sand fly species caught in the canine leishmaniasis focus of Mont-Rolland by year of collection and gender (M = Males, F = Females, T = total).

Collection Year Sand fly species	2007			2008			2009			2010			Total		
	M	F	T	M	F	T	M	F	T	M	F	T	M	F	T*%*
*Ph*. *duboscqi*	14	11	25	*	3	3	*	1	1	9	8	17	**23**	**23**	**46(1.26)**
*Ph*. *rodhaini*	2	3	5	*	1	1	*	0	0	0	2	2	**2**	**6**	**8(0.22)**
*Se*. *schwetzi*	234	322	556	*	205	205	*	220	220	128	158	286	**362**	**905**	**1267(34.7)**
*Se*. *antennata*	2	3	5	*	3	3	*	1	1	5	12	17	**7**	**19**	**26(0.7)**
*Se*. *dubia*	116	254	370	*	175	175	*	152	152	59	187	246	**175**	**768**	**943 (25.8)**
*Se*. *magna*	29	85	114	*	87	87	*	38	38	34	72	106	**63**	**282**	**34 (9.44)**
*Se*. *clydei*	24	77	101	*	15	15	*	17	17	29	30	59	**53**	**139**	**192(5.26)**
*Se*. *adleri*	23	39	62	*	10	10	*	8	8	33	31	64	**56**	**88**	**144(3.94)**
*Se*. *buxtoni*	76	84	160	*	26	26	*	29	29	253	215	468	**329**	**354**	**683(18.69)**
Total	520	878	1398	*	525	525	*	466	466	550	715	1265	**1070**	**2584**	**3654**

*in 2008 and 2009, Males have not been identified

### Distribution of the sand fly species in the different environments

**Fig 2:**
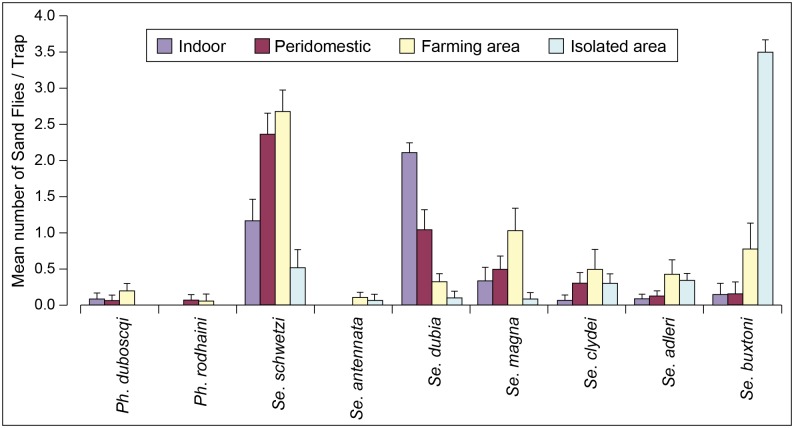
Distribution of sand fly species in the different environments. Average number of sand flies (all traps) for each species in the different sites of capture (indoor, peridomestic, farming and sylvatic area).

The complete logistic regression results are shown in supplementary data (S1 Table). *Sergentomyia schwetzi* was most commonly found in the peridomiciliary environment, and its abundance in this environment is significantly different from the three other environments (*P*-values less than 1.0E-07, (see S1 Table for details). *Sergentomyia dubia* is most commonly found in the intradomiciliary environment and in this case too, this environment is significantly different from all three other environments (*P*-values always <2.0E-16). *Sergentomyia*. *magna* was similarly distributed in the different environments. The species *Se*. *clydei*, *Ph*. *duboscqi*, *Se*. *antennata*, *Se*. *adleri* were rarely found indoors and in peridomestic areas, but and were caught mainly in farming areas (59%, 68%, 91% and 72%, respectively). *Sergentomyia buxtoni* was negatively associated with intradomiciliary-, peridomiciliary- and farming habitats, supporting its association with the sylvatic environments ((*P*-values always <2.0E-16); logistic regression analysis, supplementary data (S1 table and Fig 2).

### Leishmania promastigote infection in female sand flies

Among the captured females, 612 specimens belonging to the nine phlebotomine species were dissected and their digestive tract was examined under a microscope to detect flagellated parasites. Only four females (two *Se*. *dubia* and two *Se*. *schwetzi*) were infected. The parasite strains isolated from these four females were inoculated in culture tubes with NNN medium and flagellated forms were clearly observed after three days of culture. Molecular identification of the cultured parasites using the nested PCR-based method indicated that the four *Sergentomyia* specimens were infected by *Leishmania* species. A band of about 750pb similar to the amplicon size of the *L*. *infantum* reference (Table 2, S1 Fig) was amplified from the cultures of one *Se*. *dubia* and two *Se*. *schwetzi* specimens (SEN27, SEN19 and FR011, respectively). A band of about 800bp, matching the amplicon size of the *L*. *tarentolae* reference, was detected in the culture (SEN15) from the other *Se*. *dubia* specimen (Table 2, S1 Fig).

**Table 2:** *Leishmania* promastigote infection in female sand flies. Number of dissected females per species and number and percentage of females found to be infected by *L*. *infantum* or *L*. *tarentolae*.

Species	No. of dissected Females	No. of females infected by *L*. *infantum* (%)	No. of females infected by *L*. *tarentolae* (%)
*Ph*. *duboscqi*	2	0	0
*Ph*. *rodhaini*	1	0	0
*Se*. *schwetzi*	253	2 (0.79)	0
*Se*. *antennata*	8	0	0
*Se*. *Dubia*	246	1 (0.40)	1 (0.40)
*Se*. *Magna*	58	0	0
*Se*. *Clydei*	11	0	0
*Se*. *adleri*	10	0	0
*Se*. *buxtoni*	23	0	0
Total	612	3 (0.49)	1 (0.16)

Notes: Dissections were made only in 2008 and 2009. The *Se*. *dubia* infected with *L*. *infantum*, one of the *Se*. *schwetzi* infected with *L*. *infantum* and the *Se*. *dubia* infected with *L*. *tarentolae* were caught in 2008; The other *Se*. *schwetzi* infected with *L*. *infantum* was caught in 2009

To confirm the identifications, we sequenced the amplicons of the four isolates. All the sequences showed a high average quality that allowed a nucleotide reading between 70% and 97% of the amplicons. These sequences were submitted to GenBank and were assigned the accession numbers KU587707 to KU587710 corresponding to the following isolates, SEN15, SEN19, SEN27, FR011 respectively. BLAST analysis showed 94% homology between SEN15 sequence (KU587707) and the minicircle sequence of *L*. *tarentolae* (GeneBank accession number: AF380693.1). The SEN19 sequence (KU587708) was 93% similar to a *L*. *chagasi* (synonymous *L*. *infantum*) kinetoplast minicircle sequence (GeneBank accession number: JX156608.1), SEN27 sequence (KU587709) was 98% similar to another *L*. *chagasi* (syn. *L*. *infantum*) kinetoplast minicircle sequence (GeneBank accession number: AF308682) and FR011 99% similar to a kinetoplast minicircle sequence obtained from a sand fly isolate (GeneBank accession number: AJ270104.1) and 79% similar to a *L*. *infantum* kinetoplast minicircle sequence (GeneBank accession number: AF027577.1). These data support the conclusion that the SEN15 isolate from one *Se*. *dubia* female belonged to *L*. *tarentolae* and that the three other isolates, SEN19 isolate from *Se*. *schwezi* female and SEN27 and FR011 from *Se*. *dubia* females belonged to *L*. *infantum*.

The *Se*. *dubia* female infected with *L*. *infantum* (SEN27) was caught in a light trap inside a house in the village of Thiaye. It was not engorged and not gravid. We could distinguish a mixture of forms in which highly motile metacyclic promastigotes were clearly observed (with relatively short body length and flagellum length two times body length) in the anterior part of the midgut as described in Bates et al. [19]. Of the two *Se*. *schwetzi* females infected by *L*. *infantum* (SEN19 and FR011), one was collected in a peridomestic environment by CDC light trap in Pallo Diale. Many motile promastigotes were found in the midgut, which contained brown-colored blood corresponding to an old (36–48 hours) partially digested blood-meal. The second one was caught by indoor spraying in Khaye Diagal. This female was not blood-fed or gravid and its anterior midgut contained many metacyclic promastigotes. The *Se*. *dubia* specimen positive for *L*. *tarentolae* (SEN15) was caught indoor, in Khaye Diagal. The gut contained many promastigotes and digested blood.

### Leishmania DNA detected in sand flies

Then, 2113 females, of which 156 were blood-fed and 199 gravid, from the seven villages were screened for *Leishmania* parasite infection by using the nested kDNA PCR assay [13]. *L*. *infantum* kDNA could be amplified in 69 specimens (3.26%) (Table 3). The positive females belonged to three species: *Se*. *dubia* (29 specimens, 5.38% of all captured *Se*. *dubia*), *Se*. *schwetzi* (32 specimens, 4.19%) and *Se*. *magna* (8 specimens, 3.64%). The females from the other *Sergentomyia* species and the two *Phlebotomus* species were all negative. *L*. *tarentolae* kDNA was found in 24 specimens, mainly in *Se*. *dubia* females, the proven *L*. *tarentolae* vector in Senegal [20], but also in *Se*. *schwetzi*, *Se*. *clydei* and *Se*. *buxtoni* specimens (Table 3).

**Table 3:** PCR diagnostic results. Number of tested females and number of females infected by *Leishmania infantum* or *Leishmania tarentolae* according to the sand fly species and year. (T = Number. of tested Females, Pi = No. of females infected by *L*. *infantum*, Ps = No. of females infected by *L*. *tarentolae*).

Collection Year Sand fly species	2007			2008			2009			2010			Total		
	T	Pi	Ps	T	Pi	Ps	T	Pi	Ps	T	Pi	Ps	T	Pi (%)	Ps (%)
*Ph*. *duboscqi*	11	0	0	3	0	0	1	0	0	8	0	0	**23**	**0**	**0**
*Ph*. *rodhaini*	2	0	0	0	0	0	0	0	0	1	0	0	**3**	**0**	**0**
*Se*. *schwetzi*	269	11	0	172	7	2	185	9	0	137	5	0	**763**	**32(4.19)**	**2(0.6)**
*Se*. *antennata*	3	0	0	3	0	0	1	0	0	9	0	0	**16**	**0**	**0**
*Se*. *dubia*	179	9	4	122	4	2	107	6	4	131	10	5	**539**	**29(5.38)**	**15(2.78)**
*Se*. *magna*	67	3	0	69	2	0	30	0	0	54	3	0	**220**	**8(3.64)**	**0**
*Se*. *clydei*	68	0	2	13	0	0	16	0	0	26	0	1	**123**	**0**	**3(2.44)**
*Se*. *adleri*	32	0	0	11	0	0	7	0	0	23	0	0	**73**	**0**	**0**
*Se*. *buxtoni*	84	0	0	26	0	0	29	0	1	214	0	3	**353**	**0**	**4(1.13)**
**Total**	**715**	**23**	**6**	**419**	**13**	**4**	**376**	**15**	**5**	**603**	**18**	**9**	**2113**	**69(3.26)**	**24(1.14)**

Among the 69 females infected with *L*. *infantum*, 15 had a blood-meal and 13 were gravid. Thus, the proportion of *L*. *infantum-*positive specimens was higher among blood-fed (9.62%) and gravid (6.53%) than among unfed females (2.32%) (Table 4). It is worth noting that the positive individuals were distributed over the years of collection, supporting the fact that *Leishmania* is circulating constantly in sand flies.

**Table 4:** *Leishmania infantum*-positive females and physiological status. Details of positive females by PCR amplification according to the year and the physiological status.

Year	Species	Unfed females		Blood-fed females		Gravid females	
		T	Pi	T	Pi	T	Pi
2007	*Se*. *schwetzi*	220	5	22	3	27	3
	*Se*. *dubia*	142	6	19	2	18	1
	*Se*. *magna*	56	3	5	0	6	0
2008	*Se*. *schwetzi*	139	4	14	1	19	2
	*Se*. *dubia*	97	2	13	1	12	1
	*Se*. *magna*	56	1	6	1	7	0
2009	*Se*. *schwetzi*	151	6	15	2	19	1
	*Se*. *dubia*	85	4	11	0	11	2
	*Se*. *magna*	24	0	3	0	3	0
2010	*Se*. *schwetzi*	111	2	12	1	14	2
	*Se*. *dubia*	104	7	14	2	13	1
	*Se*. *magna*	45	1	4	2	5	0

T = total; Pi: number of females showing a PCR-band pattern equal to L. infantum.

### Sand fly infection rate and environment

The specimens identified as *L*. *infantum-*positive by PCR assay were mostly captured in areas where both humans and dogs live: indoor (33 of the 562 indoor specimens; 5.87%) and in peridomestic environments (28 of the 573 peridomestic specimens; 4.88%) (Table 5). In environments less frequented by dogs and humans during the period of sand fly activity (night and dusk), such as farming areas, only 8 females out of the 642 tested (1.25%) were infected by *L*. *infantum*. In the sylvatic area, none of the collected specimens was positive for *L*. *infantum*. Logistic regression analysis showed that the probability of infection for the species *Se*. *dubia* (*p-value* = 0.011) and *Se*. *schwetzi* (*p-value* = 0.0013) was significantly associated with the environment. Specifically, the probability of infection was higher indoor and in peridomestic environments for *Se*. *dubia* sand flies and in peridomestic areas for *Se*. *schwetzi*.

**Table 5:** Sand fly infection rate and environment. Number of tested females and number and percentage of *L*. *infantum*-positive females by PCR for each species according to the environment (indoor, peridomestic, farming and sylvatic area).

Environment Species	Indoor		Peridomestic		Farming area		Sylvatic area		
	Tested	*L*. *inf*+ ^a^ (%)	Tested	*L*. *inf*+ ^a^ (%)	Tested	*L*. *inf*+ ^a^ (%)	Tested	*L*. *inf*+ ^a^ (%)	Total
*Ph*. *duboscqi*	5	0	7	0	11	0	0	0	0
*Ph*. *rodhaini*	0	0	2	0	1	0	0	0	0
*Se*. *schwetzi*	158	9 (5.70)	265	18 (6.79)	285	5 (1.75)	55	0	32 (4.19)
*Se*. *antennata*	2	0	0	0	11	0	3	0	0
*Se*. *Dubia*	318	23 (7.23)	141	6 (4.26)	72	0	8	0	29 (5.38)
*Se*. *magna*	45	1 (2.22)	64	4 (6.25)	109	3 (2.75)	3	0	8 (3.62)
*Se*. *Clydei*	9	0	47	0	48	0	19	0	0
*Se*. *Adleri*	4	0	14	0	35	0	20	0	0
*Se*. *buxtoni*	21	0	33	0	70	0	228	0	0
**Total**	562	33 (5.87)	573	28 (4.88)	642	8 (1.25)	336	0	69 (3.27)

***L*. *inf*+**: positive females showing a PCR band pattern equal to *L*. *infantum*

### Blood-meal analysis

PCR analysis of 141 blood-meals in females from seven species using primers to amplify the AluYb8 repeat and the *PNOC* gene gave positive results in 43 samples (30.5%). Some PNOC amplicons could not be identified because the obtained sequence was too short for sequence comparison with BLAST and thus the blood-meal source was reported as “non-human mammals”.

The blood-meal analysis revealed a large variety of blood sources (Table 6). *Sergentomyia*. *dubia* blood-meals were mainly from humans. *Sergentomyia*. *schwetzi* appeared to feed on a wide range of hosts, such as humans, dogs, horses, cows and mice. For the other species, the sequencing results indicated either human blood or blood from “non-human mammals”.

**Table 6:** Results of blood-meal analysis. Number of tested blood-meals, number of identified blood-meals and origin of the blood-meal for each species based on sequence analysis of the amplicons obtained using primers that amplify the human-specific AluYb8 repeat and the mammalian *PNOC* gene.

Species	Tested blood-meals	Identified blood-meals	Origin of the blood meal (number of samples)
*Ph*. *duboscqi*	1	1	non-human mammals (1)
*Ph*. *Rodhaini*	0	0	
*Se*. *antennata*	0	0	
*Se*. *dubia*	51	21	human (18), non-human mammals (3)
*Se*. *magna*	18	1	human (1)
*Se*. *schwetzi*	52	15	human (5), non-human mammals (5), horse (1), dog (2), mouse (1), cow (1)
*Se*. *clydei*	3	1	human (1)
*Se*. *adleri*	1	0	
*Se*. *buxtoni*	13	4	human (2), non-human mammals (2)
**Total**	**141**	**43**	**horse (1), dog (2), mouse (1), cow (1)**

### Association between L. infantum prevalence in sand flies and L. infantum seroprevalence in dogs and humans

Logistic regression analysis was also employed to explore the effect of *L*. *infantum* prevalence in sand flies on the probability for dogs and humans to be seropositive for *L*. *infantum*. For each village, and for each sand fly species, the prevalence of infection by *L*. *infantum* was computed and then log-transformed. The serological status (*L*. *infantum* positive/negative) of 315 people (73 positive) and 160 dogs (74 positive) in these villages was retrieved from Faye et al. [6, 16].

**Fig 3:**
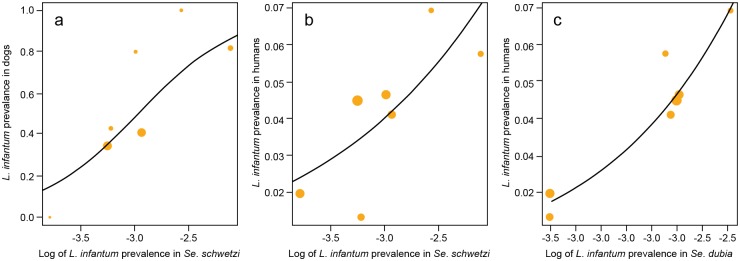
*L*. *infantum* prevalence in dogs (a) and humans (b and c) versus *L*. *infantum* prevalence in *Se*. *schwetzi* (a and b) and *Se*. *dubia* (c). Circles represent villages. The circle area is proportional to the sample size. Solid lines represent the fitted logistic regression curves.

The probability for a dog to be seropositive for *L*. *infantum* was strongly correlated with *L*. *infantum* prevalence in *Se*. *schwetzi* in the dog's village. The odds ratio (OR) associated with a 10% increase of prevalence was 8.5, 95% CI (1.88–35.3), *p*-value = 0.0041 (Fig 3A). There was no association with *L*. *infantum* prevalence in the other sand fly species.

The probability of a human being seropositive for *L*. *infantum* was correlated with *L*. *infantum* prevalence in *Se*. *schwetzi* in the seven villages. The OR associated with a 10% increase of prevalence was 1.96, 95% CI (1.16–3.31), *p*-value = 0.012 (Fig 3B). The probability of a human being infected was also correlated with *L*. *infantum* prevalence in *Se*. *dubia* in the village [OR = 7.65, 95% CI = (2.15–27.16), *p*-value = 0.0013] (Fig 3C). There was no association with *L*. *infantum* prevalence in the other sand fly species.

## Discussion

The main goal of this study was to identify the sand fly vectors of *L*. *infantum* in Senegal. Several previous epidemiological studies showed that *L*. *infantum* transmission in Mont-Rolland is endemic and well established in dogs and humans [6, 16]. Nevertheless, despite the extensive research of the past 45 years, the *L*. *infantum* vectors in this focus of canine leishmaniasis are still unknown [4, 5, 9]. This could be explained by transmission via an unusual vector [12]. In the Old World, *L*. *infantum* is classically transmitted by bites of female sand flies belonging to the *Phlebotomus* genus and to the *Larroussius* and, secondarily, the *Adlerius* subgenera [2, 19–22]. In Senegal these species are absent. Among the 30 sand fly species currently recorded in Senegal, 27 belong to the *Sergentomyia* genus and three to the *Phlebotomus* genus [*Ph*. *duboscqi* and *Ph*. *bergeroti* (subgenus *Phlebotomus*), and *Ph*. (*Anaphlebotomus*) *rodhaini*] [12].

According to Killick-Kendrick [2], the essential criteria that must be met to consider a sand fly species a proven vector of human or canine leishmaniasis are: i) anthropophily and/or attraction to dogs; and ii) repeated isolation and identification of the same *Leishmania* in sand flies and in humans and/or dogs. The supporting observations are: iii) demonstration that the incriminated sand fly commonly feeds on the reservoir host(s); iv) presence of the sand fly species in places where the *Leishmania* and the caused disease are found; v) demonstration that the sand fly species can support the flourishing development of the parasite; and vi) demonstration that the sand fly species can transmit the parasite by bite [2]. These criteria were updated by Ready in 2013. Criterion 1 described in Ready [23] deals with the isolation and/or typing of promastigotes from several unambiguously identified wild female flies not containing recent blood meals (less than 36 h old) on more than one occasion. Criterion 2 specifies that infective (metacyclic) forms of *Leishmania* should be seen in the anterior midgut and on the stomodeal valve of naturally infected female flies. Concerning Criterion 3, the fly species under study must be attracted to and readily bite humans and any reservoir host. Criterion 4 deals with the evidence of a strong ecological association between flies, humans and any reservoir hosts and Criterion 5 concerns the demonstration of the experimental transmission. The two last criteria 6 and 7 are based on mathematical modeling of transmission and of links between disease prevalence and biting.

In the present work, we based our approach on several of these criteria to identify the *L*. *infantum* vectors in the Mont-Rolland community.

### Species and environment: endophily/exophily, feeding behavior

In this study, almost all the sand fly specimens captured around the habitats of dogs and humans belonged to the *Sergentomyia* genus. Indeed, the two *Phlebotomus* species were poorly represented (*Ph*. *rodhaini*: 8 specimens, 0.22%; *Ph*. *duboscqi*: 46 specimens, 1.26% of all specimens). *Phebotomus duboscqi* sand flies were caugh mainly in farming areas in the sandy ecosystem, where canine leishmaniasis is less frequent [6, 10], Fig 1). Concerning *Ph*. *rhodlaini*, a previous study showed that this species is probably underestimated because of unsuitable traps and could play a role in *L*. *donovani* transmission between animal reservoir hosts [24]. Rodent or dog-baited traps were not used in this study but light traps were placed above and in rodent burrows, around dogs in farming and peridomestic areas and we collected all residual fauna (all the insects staying inside after night) indoor. It is thus likely that the low number of *Ph*. *rhodaini* collected reflect the population of this species in the environments where *L*. *infantum* is transmitted.

*Sergentomyia dubia*, *Se*. *schwetzi*, and to a lesser extent *Se*. *magna*, were the most abundant species captured indoors and in peridomestic environments, around infected dogs and serologically positive humans. As previously reported [11], *Se*. *dubia*, the vector of the gecko leishmaniasis in Senegal, showed the most endophilic behavior. This species was significantly associated with the indoor environment where the majority of blood-fed and gravid females were also caught. Blood-meal analysis confirmed that *Se*. *dubia* feeds frequently on humans (Table 6). These results were unexpected because previous studies associated its presence indoors with its preference for reptiles, and particularly for geckos [25]. Nevertheless, this is consistent with the strong adaptability of this species [11, 25]. *Sergentomyia schwetzi*, the most abundant species captured, was found predominantly outdoors (peridomestic and farming environments), although it was also well represented indoors. Blood-fed and gravid females were more numerous in peridomestic and farming areas, suggesting a more exophilic behavior. In accordance with previous studies [25], the results of the blood-meal source analysis (often humans and dogs) confirmed this opportunistic behavior [11, 25]. *Sergentomyia magna* was present in the different sites of capture. Although slightly more abundant in farming areas, its presence in peridomestic areas and indoors was not negligible, suggesting a regular contact with dogs and humans. One of the *Se*. *magna* females had fed on humans.

The other *Sergentomyia* species were very rare or almost absent indoors and in peridomestic areas, although the blood-meals of one *Se*. *clydei* and two *Se*. *buxtoni*, caught indoor, were of human origin. *Sergentomyia clydei*, *Se*. *antennata* and *Se*. *adleri* were most abundant in farming areas. In agreement with previous studies [9, 12, 25], *Se*. *buxtoni* was mainly collected in the sylvatic area, suggesting a very exophilic behavior and feeding preferences focused on wild animals.

### Leishmania infection

The finding of naturally infected sand flies is essential to incriminate a vector and also to study the infection rates in endemic areas [2, 19, 21]. In the present study, microscopic examination indicated that 0.4% of *Se*. *dubia* and 0.79% of *Se*. *schwetzi* were infected with living *L*. *infantum* promastigotes of which two nongravid and unfed individuals (one *S*. *schwetzi* and one *S*. *dubia*) had mature forms in the anterior midgut. Natural infections in unfed specimens strongly suggest that the parasites have overcome the main barriers to metacyclogenesis (i.e., the digestive enzymes and the peritrophic membrane) in the sand fly midgut [21]. Consequently, the mature and metacyclic promastigotes observed in unfed and non-gravid females correspond more likely to infective forms. To our knowledge, these results are the first report of natural infection of *Sergentomyia* species by *L*. *infantum*. It is consistent with the prevalence of *L*. *infantum* infection in many sand fly vectors [26–29]. As the microscopic detection of promastigotes in dissected flies is difficult to carry out, the prevalence rates reported using this method are generally low (0.01–1%) even in competent vectors and in endemic areas [2, 27]. Therefore, the isolation of *L*. *infantum* by dissection in *Se*. *dubia* and *Se*. *schwetzi* individuals is an important finding.

The results of the nested PCR diagnostic assay confirmed the dissection data. They revealed the presence of *L*. *infantum* kDNA in *Se*. *dubia* (5.38% of all tested specimens for this species), in *Se*. *schwetzi* (4.19%) and also in *Se*. *magna* (3.64%). These percentages are in agreement with the infection rates reported in proven vectors by Aransay *et al*. [21] and Kishor *et al*. [30]. Nevertheless, it is essential to keep in mind that the detection of *Leishmania* DNA in a sand fly does not prove the vector competence [31, 32]. Indeed, a positive PCR result does not allow establishing whether the *Leishmania* kDNA was due to *Leismania* ingested while feeding, or to the presence of well-established or developing parasites [21, 29, 30, 32]. In the current study, the percentage of PCR-positive sand flies was significantly different in blood-fed (9.62%), gravid (6.53%) and unfed and non-gravid females (2.32%). The PCR-positive unfed/non-gravid females (2.74% of *Se schwetzi*, 4.44% of *Se*. *dubia* and 2.76% of *Se*. *magna*) may reflect the survival of parasites (or persistence of DNA) in these sand fly species. Indeed, the presence of *Leishmania* kDNA in these females strongly suggests that the parasites were ingested several days before the capture and that they had started their developmental cycle. However, we cannot exclude the persistence of DNA without any infective role, especially because the sand flies were caught in the vicinity of infected dogs. Nevertheless, the microscopic observation of mature *L*. *infantum* promastigotes in unfed females strongly suggests that they are not only carrying parasite DNA, but that they could be competent vectors. None of the other species was found to be infected with *L*. *infantum* both by dissection and PCR testing.

### Possible vectors of L. infantum in Mont-Rolland and transmission

Currently, phlebotomine sand flies remain the exclusive proven vectors of leishmaniasis, although other insects, such as midges, are suspected to act as leishmaniasis vectors [33] and few cases of vertical transmission (mother to child) have been reported [34, 35] The *Sergentomyia* genus is still not considered to be involved in leishmaniasis transmission [35]; however, several studies have detected *Leishmania* DNA and in one of them live parasites of *L*. *major* in various *Sergentomyia* and *Spelaeomyia* species, suggesting that this genus could play a role in *Leishmania* transmission [31, 36–40]

Our working hypothesis was that the vector species should belong to the *Phlebotomus* genus, which contains the classical vectors of *L*. *infantum* in the Old World. However, in the Mont-Rolland area, the two species belonging to the *Phlebotomus* genera (*Ph*. *duboscqi* and *Ph*. *rodhaini)* were not abundant and not infected by *L*. *infantum* parasites. These results are in agreement with previous works showing that *Ph*. *duboscqi*, the main *L*. *major* vector, is refractory to infection by *L*. *infantum* [41].

Conversely, some *Sergentomyia* species satisfied several criteria for vector incrimination. First, *Se*. *dubia*, *Se*. *schwetzi* and, to a lesser extent, *Se*. *magna* were the most abundant species captured indoors and in peridomestic environments surrounding infected dogs and serologically positive humans. *Sergentomyia dubia* showed an endophilic and anthropophilic behavior, feeding frequently on humans and *Se*. *schwetzi* a more exophilic and opportunistic behavior. *Sergentomyia magna* was moderately collected in the different sites, but displayed also a regular contact with dogs and humans. *Sergentomyia dubia* and *Se*. *schwetzi* females were found to be infected with *Leishmania* parasites both by dissection and by PCR-based diagnostic assay. *Se*. *magna* was found to be infected with *Leishmania* only by PCR. The isolates obtained from the dissections were successfully cultured and characterized as *L*. *infantum*, similarl to the parasites isolated from the dogs in this region [16]. Furthermore, unfed and non-gravid females were also infected, strongly suggesting that *L*. *infantum* survived after digestion of the blood-meal or egg laying. These data suggest that *L*. *infantum* can develop in *Se*. *dubia* and in *Se*. *schwetzi*. Nevertheless, a recent experimental study on the susceptibility of *Se*. *schwetzi* to *L*. *donovani*, *L*. *infantum* and *L*. *major* reported that this sand fly species is refractory to infection by *L*. *infantum* because the parasites were defecated with the blood remnants [42]. It is essential to underline that the conditions in natural populations and in laboratory setups can be quite different. Moreover, for a rigorous analysis of the *Sergentomyia* capacity to transmit *Leishmania*, it is important to work with parasites and sand flies collected from the area in which the transmission is suspected. Indeed, the sand fly-*Leishmania* interactions could be the results of a specific and recent co-evolution in that area and the ability of *Sergentomyia* species to transmit *L*. *infantum* could be region-specific. On the other hand, in the study by Sadlova et al. [42], the *Se*. *schwetzi* colony was established from specimens collected in north-western Ethiopia and the *L*. *infantum* strain was isolated in Turkey from a sand fly specimen belonging to the *Ph*. *tobbi* species [42]. No experimental data are available either on *Se*. *dubia* or *Se*. *magna*.

Our hypothesis is also supported by the results of the statistical analyses that demonstrate a strong ecological association between flies, humans and hosts (criteria 4 of Ready, [23]. Indeed, *Se*. *dubia* and *Se*. *schwetzi* were strongly associated with humans and dogs (indoor and peridomestic environments); the probability of infection was higher indoors and in peridomestic environments for *Se*. *dubia* sand flies and in peridomestic areas for *Se*. *schwetzi;* the PCR-positive *Se*. *schwetzi* and *Se*. *dubia* specimens were significantly correlated with the seroprevalence data in dogs and/or humans.

Taken together, these data strongly suggest, for the first time, that *Se*. *dubia* and *Se*. *schwetzi* are possible vectors of canine leishmaniasis and responsible for the contact between the Leishmania parasites and humans in the Mont-Rolland area. This study challenges one more time the dogma claiming that Phlebotomus is the only genus responsible for Leishmania transmission in the Old World. Thus, these findings should be confirmed by experimental infection using Se. schwetzi and Se. dubia colonies and L. infantum parasites from the Mont-Rolland community.

## Supporting Information

S1 Fig Representative results of the nested PCR-based identification of *Leishmania* strains isolated from female sand flies. A) Reference strains: 1 = negative control, 2 = *Leishmania infantum* (ITMAP 263), 3 = *L*. *major* (5ASKH), 4 = *L*. *tropica* (K27), 5 = *L*. *tarentolae*. B) 1 = *L*. *infantum* (ITMAP 263), 2 = *Sergentomyia dubia* female infected by *L*. *tarentolae*, 3 = *Se*. *schwetzi* female infected by *L*. *infantum*, 4 = *Se*. *dubia* female infected by *L*. *infantum*, 5 = negative control (no template).
(TIF)

S1 Table Results of logistic regression of the proportion of captures from a given species on the environment type. Only the *Sergentomyia* species were studied, the *Phlebotomus* captures being too rare. The “Isolated” environment was taken as the baseline. The effect of the environment is measured in terms of odds-ratio (OR) for the proportion of captures of the studied species. The *P-*values correspond to the test of the null hypothesis OR = 1. Post-hoc tests were conducted, where the hypotheses tested are equality of two odds-ratios.
(DOCX)
