# Supplementary figures and images for: Transmission of Leishmania infantum in the Canine Leishmaniasis Focus of Mont-Rolland, Senegal: Ecological, Parasitological and Molecular Evidence for a Possible Role of Sergentomyia Sand Flies
Source: PLoS Negl Trop Dis. 2016 Nov 2;10(11):e0004940. doi: 10.1371/journal.pntd.0004940 (PMC5091883; doi:10.1371/journal.pntd.0004940)

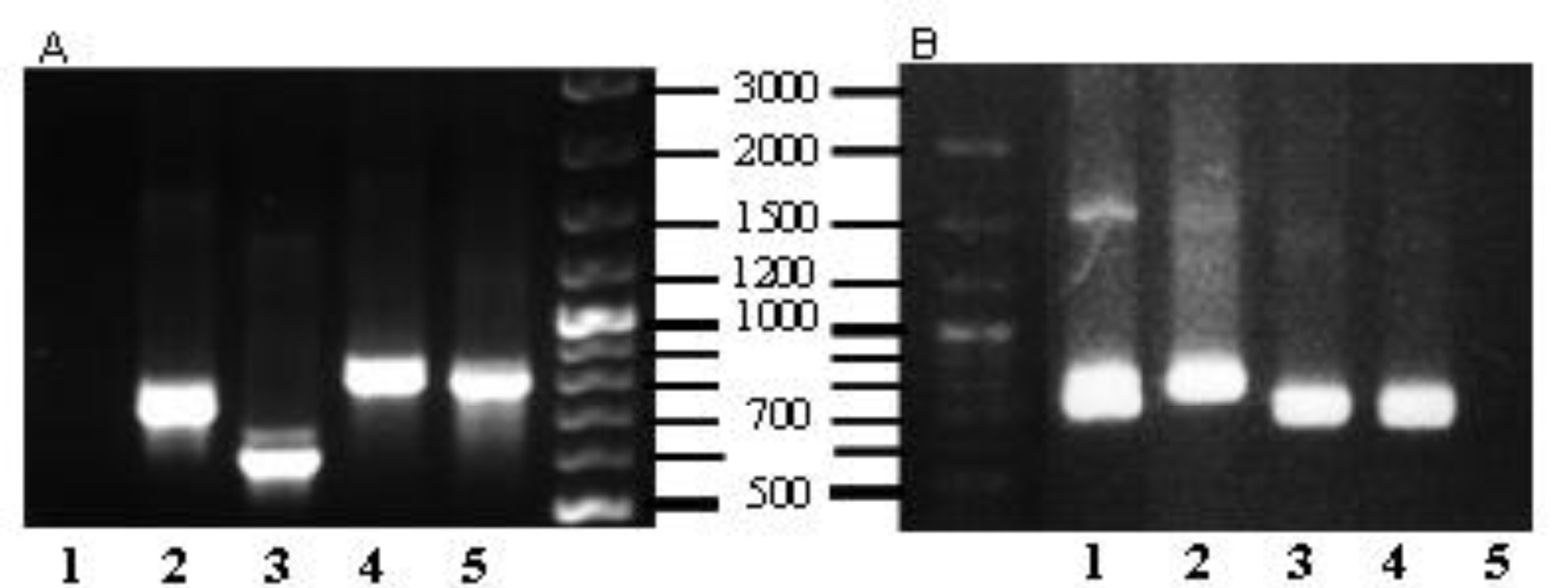

Supplement: S1 Fig — A) Reference strains: 1 = negative control, 2 = Leishmania infantum (ITMAP 263), 3 = L. major (5ASKH), 4 = L. tropica (K27), 5 = L. tarentolae. B) 1 = L. infantum (ITMAP 263), 2 = Sergentomyia dubia female infected by L. tarentolae, 3 = Se. schwetzi female infected by L. infantum, 4 = Se. dubia female infected by L. infantum, 5 = negative control (no template). (TIF) [file pntd.0004940.s001.tif]
