# Supplementary material for: Transmission of Leishmania infantum in the Canine Leishmaniasis Focus of Mont-Rolland, Senegal: Ecological, Parasitological and Molecular Evidence for a Possible Role of Sergentomyia Sand Flies
Source: PLoS Negl Trop Dis. 2016 Nov 2;10(11):e0004940. doi: 10.1371/journal.pntd.0004940 (PMC5091883; doi:10.1371/journal.pntd.0004940)
Supplement: S1 Table — Only the Sergentomyia species were studied, the Phlebotomus captures being too rare. The “Isolated” environment was taken as the baseline. The effect of the environment is measured in terms of odds-ratio (OR) for the proportion of captures of the studied species. The P-values correspond to the test of the null hypothesis OR = 1. Post-hoc tests were conducted, where the hypotheses tested are equality of two odds-ratios. (DOCX) [file pntd.0004940.s002.docx]

**Supplementary Table 1.**

Results of logistic regression of the proportion of captures from a given species on the environment type. Only the *Sergentomyia* species were studied, the *Phlebotomus* captures being too rare. The “Isolated” environment was taken as the baseline. The effect of the environment is measured in terms of odds-ratio (OR) for the proportion of captures of the studied species. The *P-*values correspond to the test of the null hypothesis OR=1. Post-hoc tests were conducted, where the hypotheses tested are equality of two odds-ratios.

| Parameters | Estimated value | 95% conf. interval | *P*-value |
| --- | --- | --- | --- |
| *Sergentomyia schwetzi* | | | |
| Farming | OR=3.14 | 2.49 - 3.94 | <2.0E-16 |
| Peridomiciliar | OR=4.99 | 3.97 - 6.27 | <2.0E-16 |
| Intradomiciliar | OR=1.39 | 1.08 - 1.80 | 0.042 |
| test peri = intra |  |  | <2.0E-16 |
| test peri = farming |  |  | 2.1E-06 |
| test intra = farming |  |  | 6.4E-12 |
| *Sergentomyia dubia* | | | |
| Farming | OR=3.91 | 2.41 - 6.33 | 5.9E-08 |
| Peridomiciliar | OR=10.58 | 6.68 - 16.75 | <2.0E-16 |
| Intradomiciliar | OR=55.50 | 35.14 - 87.66 | <2.0E-16 |
| test peri = intra |  |  | <2.0E-16 |
| test peri = farming |  |  | 1.4E-13 |
| test intra = farming |  |  | <2.0E-16 |
| *Sergentomyia antennata* | | | |
| Farming | OR=2.11 | 0.82 - 5.41 | 0.34 |
| Peridomiciliar | OR=0.13 | 0.016 - 1.11 | 0.19 |
| Intradomiciliar | OR=0.16 | 0.019 - 1.34 | 0.27 |
| test peri = intra |  |  | 1.00 |
| test peri = farming |  |  | 0.033 |
| test intra = farming |  |  | 0.054 |
| *Sergentomyia magna* | | | |
| Farming | OR=35.4 | 13.0 - 96.0 | 4.9E-12 |
| Peridomiciliar | OR=23.3 | 8.53 - 63.5 | 1.6E-09 |
| Intradomiciliar | OR=19.25 | 7.00 - 52.97 | 2.0E-08 |
| test peri = intra |  |  | 0.62 |
| test peri = farming |  |  | 0.0094 |
| test intra = farming |  |  | 0.00031 |
| *Sergentomyia clydei* | | | |
| Farming | OR=2.32 | 1.62 - 3.32 | 0.000017 |
| Peridomiciliar | OR=1.17 | 0.79 - 1.74 | 0.85 |
| Intradomiciliar | OR=0.25 | 0.13 - 0.49 | 0.00015 |
| test peri = intra |  |  | 6.5E-06 |
| test peri = farming |  |  | 0.00010 |
| test intra = farming |  |  | 1.3E-12 |
| *Sergentomyia adleri* | | | |
| Farming | OR=1.00 | 0.67 - 1.48 | 1.00 |
| Peridomiciliar | OR = 0.43 | 0.26 - 0.70 | 0.0034 |
| Intradomiciliar | OR=0.16 | 0.0074 - 0.34 | 0.000055 |
| test peri = intra |  |  | 0.059 |
| test peri = farming |  |  | 0.0021 |
| test intra = farming |  |  | 3.7E-06 |
| *Sergentomyia buxtoni* | | | |
| Farming | OR=0.063 | 0.049 - 0.081 | <2.0E-16 |
| Peridomiciliar | OR=0.018 | 0.012 - 0.027 | <2.0E-16 |
| Intradomiciliar | OR=0.020 | 0.013 - 0.030 | <2.0E-16 |
| test peri = intra |  |  | 0.98 |
| test peri = farming |  |  | 3.0E-09 |
| test intra = farming |  |  | 1.8E-07 |
